# Auditory pre-experience modulates classification of affect intensity: evidence for the evaluation of call salience by a non-human mammal, the bat *Megaderma lyra*

**DOI:** 10.1186/1742-9994-10-75

**Published:** 2013-12-16

**Authors:** Hanna B Kastein, Vinoth AK Kumar, Sripathi Kandula, Sabine Schmidt

**Affiliations:** 1 Institute of Zoology, University of Veterinary Medicine Hannover Foundation, Bünteweg 17, Hannover 30559, Germany; 2 School of Biological Sciences, Madurai Kamaraj University, Madurai 625021, India

**Keywords:** Bats, Acoustic communication of emotions, Affect intensity, Social call perception, Habituation-dishabituation paradigm

## Abstract

**Introduction:**

Immediate responses towards emotional utterances in humans are determined by the acoustic structure and perceived relevance, i.e. salience, of the stimuli, and are controlled via a central feedback taking into account acoustic pre-experience. The present study explores whether the evaluation of stimulus salience in the acoustic communication of emotions is specifically human or has precursors in mammals. We created different pre-experiences by habituating bats (*Megaderma lyra*) to stimuli based on aggression, and response, calls from high or low intensity level agonistic interactions, respectively. Then we presented a test stimulus of opposite affect intensity of the same call type. We compared the modulation of response behaviour by affect intensity between the reciprocal experiments.

**Results:**

For aggression call stimuli, the bats responded to the dishabituation stimuli independent of affect intensity, emphasising the attention-grabbing function of this call type. For response call stimuli, the bats responded to a high affect intensity test stimulus after experiencing stimuli of low affect intensity, but transferred habituation to a low affect intensity test stimulus after experiencing stimuli of high affect intensity. This transfer of habituation was not due to over-habituation as the bats responded to a frequency-shifted control stimulus. A direct comparison confirmed the asymmetric response behaviour in the reciprocal experiments.

**Conclusions:**

Thus, the present study provides not only evidence for a discrimination of affect intensity, but also for an evaluation of stimulus salience, suggesting that basic assessment mechanisms involved in the perception of emotion are an ancestral trait in mammals.

## Introduction

The acoustic communication of emotions may reach back beyond the evolution of human language [1,2] and constitute an ancestral mammalian trait (e.g. [3]). First, emotions are vocally expressed across mammals (for review see e.g. [4,5]) via a similar vocal apparatus and comparable physiological mechanisms governing call production (e.g. [6,7]). Second, to perceive, and to show immediate responses to, the emotions expressed in vocalisations accompanying the behaviour of a conspecific may be adaptive for both interaction partners, i.e. increase their fitness, particularly from the perspective of influencing-others scenarios of communication (e.g. [8-13]). The present paper aims at establishing evidence for adaptive behavioural responses to vocally conveyed emotions by bats, a taxon which split early from other lineages of mammals [14-16] and is of particular interest for comparative communication research (e.g. [17]).

In influencing-others scenarios, vocalisations expressing different emotions differ in their potential to disrupt the ongoing behaviour of a conspecific, and to re-direct its attention to assess the sound source. Thus, these distraction effects may serve as indicators of perceived emotion, validated by the behavioural display and changes in its intensity [18], in playback experiments. Graded distraction effects can be expected for vocalisations expressing different intensities of emotion within a behavioural context. In various mammalian taxa, spontaneous playbacks of vocal stimuli from behaviourally validated situations of high, versus low, affect intensity evoked strong, versus weak, distraction effects, respectively, as indicated by e.g. the duration of interruption of the previously shown behaviour (carnivores [19], primates [20], rodents [21,22]), or the duration of attention towards the vocal stimulus (artiodactyls [23], primates [24], rodents [25,26]). As stimulus structure and affect intensity are necessarily linked in situation-specific vocalisations, however, the above experiments cannot differentiate to what extent the behavioural response to a stimulus is directly shaped by its acoustic structure, or is modulated by assessment mechanisms of the brain that are dependent on pre-experience, i.e. reflects the perceived emotion (for definition see [18]).

In humans, studies modulating auditory pre-experience within the experiment provided evidence for a pre-attentive evaluation of emotional speech (e.g. [27]), as well as non-speech stimuli (e.g. [28-31]) independent of direct effects of stimulus structure. Stimuli of identical acoustic structure evoked larger event-related potentials, if they were associated with an emotional, versus a neutral, connotation [28], or if the previously experienced standard stimuli were neutral, rather than emotional (e.g. [31]). In both experiments, the perceived relevance, or salience, of the stimulus rather than its acoustic structure created response asymmetries.

To study effects of stimulus salience behaviourally, habituation-dishabituation experiments (e.g. [32]) are a promising approach, because this paradigm creates a specific acoustic pre-experience during habituation and makes use of distraction effects to indicate the perception of a novel stimulus class. Indeed, habituation-dishabituation experiments have revealed that non-human mammals may categorise stimuli of graded acoustic structure as separate classes (e.g. [33,34]) and that stimuli reflecting an increase in affect intensity may be perceived as a novel class [35]. A reciprocal habituation-dishabituation design in which the stimulus class presented for habituation in one experiment is used for dishabituation in the second experiment, and vice versa, permits to compare effects of emotional pre-experience to high, and low, affect intensity stimuli. Reciprocal designs have been occasionally applied to reveal differences in the perceived relevance of infant-directed versus adult-directed speech in human infants [36], and of different call types in non-human mammals [37-39]. However, a reciprocal design has not yet been used to assess whether mammals evaluate the affect intensity in vocal stimuli. The present paper uses this design to explore whether prior exposure to acoustic stimuli of high, or low, affect intensity is able to modulate stimulus classification in a bat model.

The Indian False Vampire bat, *Megaderma lyra*, is ideally suited to address this question. First, social interactions in this species are typically accompanied by multi-syllabic, hierarchically composed calls which are specific for the behavioural situation, but also show a considerable variability within a given call type [17]. Second, this variability reflects the emotional state of the bat. In agonistic interactions about common perch use, *M. lyra* reliably expressed affect intensity, as determined by the intensity of the behavioural displays, in the acoustic parameters of the accompanying aggression, and response, calls [40]. Third, the species has been shown to discriminate differences in acoustic structure within a call type [41,42]. Finally, *M. lyra* frequently uses a sit-and-wait strategy for foraging, starting from a perch to glean prey from surfaces [43,44].

**Figure 1:**
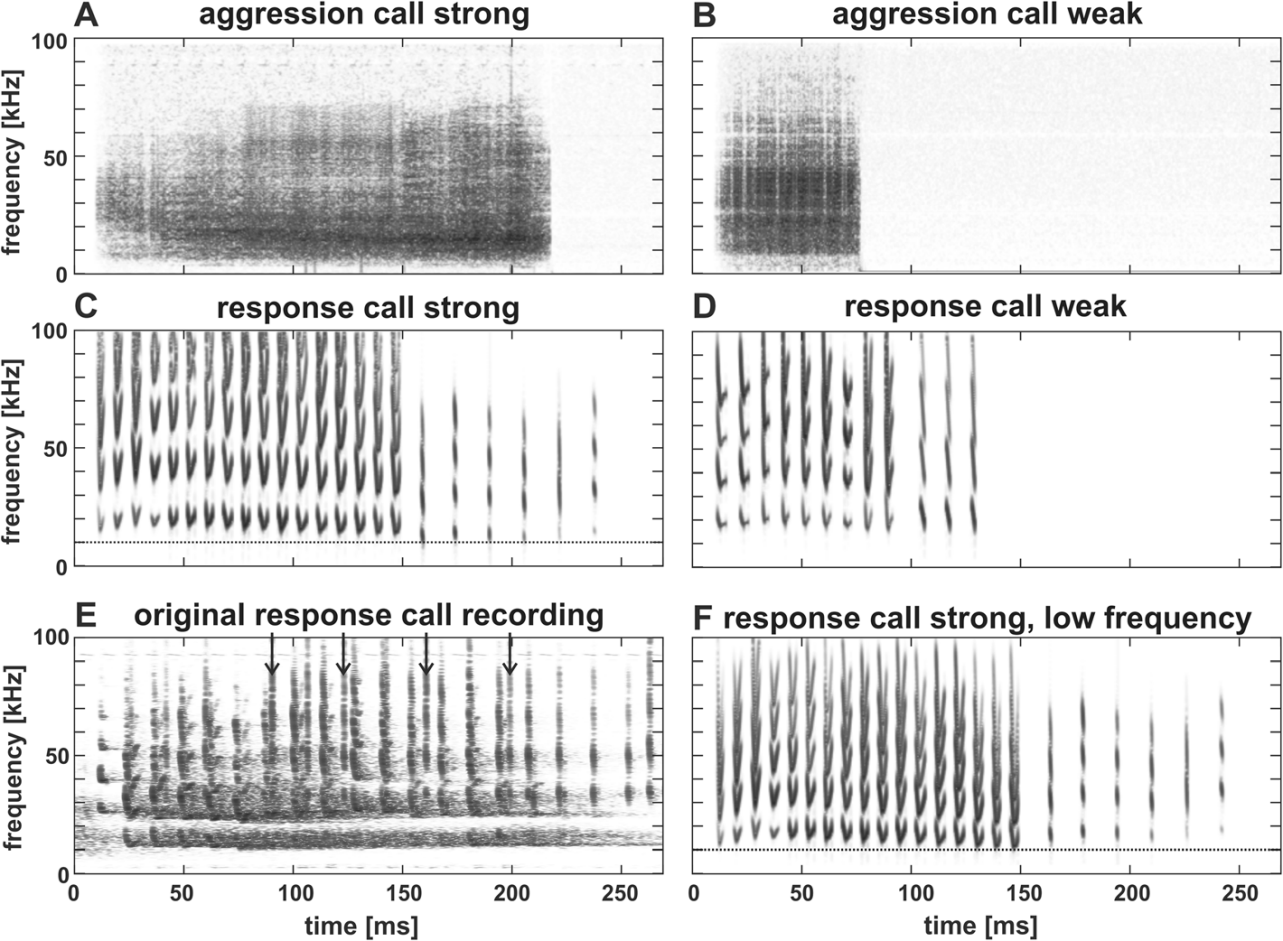
**Spectrograms representing examples for aggression (A, B), and response (C, D), call stimuli of different affect intensity.** Calls of high affect intensity are termed strong, while calls of low affect intensity are termed weak. Exemplars in **A**, **B**, **C**, and **D** were used as test stimuli in reciprocal experiments. **E** represents an original recording of a strong response call; please note interfering echolocation calls from the interaction partner (for examples see arrows), disturbing echoes and a recording artefact at about 92 kHz. **F** shows the spectrogram of the control stimulus of low frequency. The dotted line in **C** and **F** at 10 kHz is given to make the frequency shift between the respective response call stimuli more obvious.

In the present study, we exploited this foraging strategy and trained the bats to wait at a perch and focus their attention on a feeder, opening at irregular intervals. Between two food uptakes, we performed playback experiments to investigate the distraction by acoustic stimuli. Based on aggression, and response, calls of validated affect intensity [40], habituation with stimuli typical for high, or low, affect intensity (Figure 1) served to create a different emotional pre-experience. We expected (I) that the bats were able to classify stimuli of a given call type according to affect intensity. If stimulus classification was independent of emotional pre-experience, we would expect (II) that the response behaviour depended only on the difference in acoustic structure between habituation stimuli and the test stimulus, resulting in symmetric response behaviour in the reciprocal experiments. On the contrary, if stimulus classification depended on emotional pre-experience, i.e. the evaluation of stimulus salience, we would predict (III) an asymmetry in response behaviour. Specifically, we expected a release from habituation to the test stimulus of high affect intensity after experiencing stimuli of low affect intensity during habituation (experiment “weak to strong”), in contrast to the expected transfer of habituation in the reciprocal experiment (“strong to weak”). A transfer of habituation may indicate that the pre-experience with stimuli of high affect intensity resulted in a general over-habituation to the respective call type. In this case, we would predict (IV) a comparable transfer of habituation in a control experiment (“strong to low frequency”), in which bats were habituated with stimuli of high affect intensity, and tested with a control stimulus differing in syllable peak frequencies, mimicking a call from a different individual.

The present paper provides evidence for effects of emotional pre-experience and stimulus structure on auditory classification. We apply a model originally addressing auditory processing in humans which integrates the effects of vocalisation structure and auditory pre-experience to account for the response behaviour of the bats. Our results suggest an evaluation of the emotional salience of communication calls by a non-human mammal.

## Results

Twelve bats completed the playback experiments. All individuals responded with a body turn away from the feeder in the pre-test preceding the habituation-dishabituation experiments, in which the four test stimuli used in the reciprocal experiments and the response call control stimulus were played back once to test for spontaneous distraction effects. Looking times did not differ significantly for the five stimuli (test stimuli: aggression call of low affect intensity 2.4 (1.5 – 2.8) s, aggression call of high affect intensity 2.3 (2.2 – 3.2) s, response call of low affect intensity 2.0 (1.6 – 2.3) s, response call of high affect intensity 2.0 (1.4 – 2.9) s, control stimulus 2.0 (1.6 – 2.9) s; Friedman Anova, n = 12, df = 4, chi^2^ = 3.085, p = 0.54) and for the order of presentation (grand median 2.2 (2.0 – 2.3) s; Friedman Anova, n = 12, df = 4, chi^2^ = 2.422, p = 0.66).

**Figure 2:**
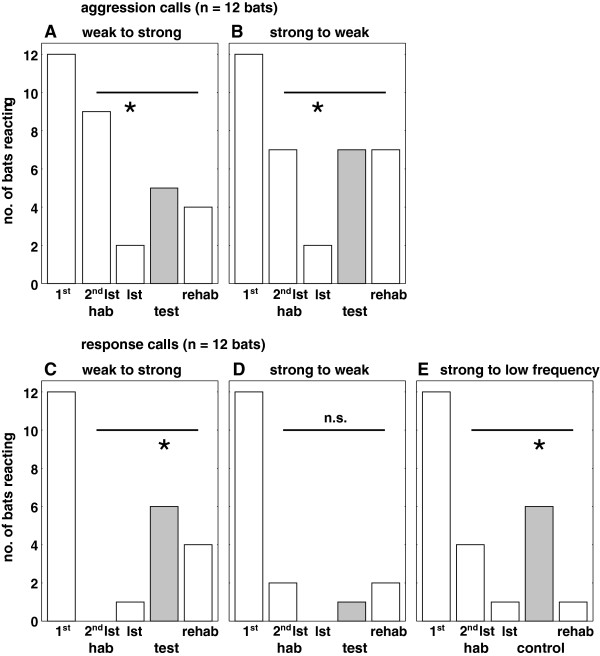
**Number of bats reacting with body turns to stimuli in the playback experiments.** Responses (no. of bats reacting) determined in the video analysis for reciprocal aggression (**A**, experiment “weak to strong”; **B**, experiment “strong to weak”) and response (**C**, experiment “weak to strong”; **D**, experiment “strong to weak”) call experiments, and the control experiment with a strong control stimulus of low frequency (**E**, experiment “strong to low frequency”) are given to the first habituation stimulus (1^st^ hab), the second to last habituation stimulus (2^nd^ lst hab), the last habituation stimulus (lst hab), the test stimulus (test/ control, highlighted in grey) and re-habituation stimulus (rehab). Across the stimuli marked by the horizontal bar, the numbers of bats reacting were compared to check for a classification according to affect intensity. An asterisk indicates that the number of bats reacting to the respective stimulus differed significantly compared to the number of bats reacting to the other three stimuli according to a Cochran’s Q test, followed by its subset comparison supplement [45], in a given experiment; “n.s.” above the bar indicates that the number of bats reacting to the four stimuli did not differ significantly according to Cochran’s Q test.

In all habituation-dishabituation experiments, the number of bats reacting decreased significantly from the first stimulus to the second to last, and last, habituation stimulus (aggression call stimuli: experiment “weak to strong”, Cochran’s Q test, n = 12, df = 2, Q = 14.36, p = 0.0008, Figure 2A; experiment “strong to weak”, n = 12, df = 2, Q = 15.0, p = 0.0006, Figure 2B; response call stimuli: experiment “weak to strong”, Cochran’s Q test, n = 12, df = 2, Q = 17.64, p = 0.000015, Figure 2C; experiment “strong to weak”, n = 12, df = 2, Q = 20.67, p = 0.000033, Figure 2D; experiment “strong to low frequency”, n = 12, df = 2, Q = 17.64, p = 0.00015, Figure 2E). The number of stimuli needed for habituation differed between experiments (Friedman Anova, n = 12, df = 4, chi^2^ = 15.78, p = 0.0033) with significantly higher numbers for aggression calls (grand median = 9.25 (8.25 - 11.25)) than for response calls (grand median = 5 (3 – 6); Fisher permutation test, n = 12, p = 0.00024).

### Discrimination of affect intensity in aggression call stimuli?

The number of aggression call stimuli needed for habituation did not depend on the experiment (“weak to strong” 8.5 (6.75 – 11.5) stimuli, “strong to weak” 9.5 (7 – 12.25) stimuli; Fisher permutation test, n = 12 bats, p = 0.22), or on experimental order (first experiment 8.5 (7 – 11.5) stimuli, second experiment 9.5 (6.5 – 12.25) stimuli; Fisher permutation test, n = 12, p = 0.52). The number of bats reacting in the reciprocal experiments is given in Figure 2A, B. The video analysis revealed that some bats showed a minute body turn not detectable by direct observation during the experiment to the second to last, or last, habituation stimulus. The number of bats reacting to the second to last habituation stimulus, the last habituation stimulus, the test stimulus and the re-habituation stimulus differed significantly in experiment “weak to strong” (Cochran’s Q test, n = 12, df = 3, Q = 8.67, p = 0.034), and showed a trend in the reciprocal experiment (Cochran’s Q test, n = 12, df = 3, Q = 6.82, p = 0.078). These differences could be attributed to the low number of bats reacting to the last habituation stimulus (n = 2 bats) and did not reflect an increased number of bats reacting to the test stimulus in both experiments (subset comparison supplements of Cochran’s Q test for experiment “weak to strong”: n = 12, df = 1, Q_diff-last_ = 4, p < 0.05; n = 12, df = 1, Q_diff-test_ = 0, n.s.; for experiment “strong to weak”: n = 12, df = 1, Q_diff-last_ = 6.82, p < 0.01; n = 12, df = 1, Q_diff-test_ = 0.76, n.s.). Thus both experiments revealed habituation to a set of aggression call exemplars, however did not support the assumption that aggression calls of high and low affect intensity were discriminated as separate classes.

The direct comparison of responses for bats reacting differently to the two test stimuli (n = 6) in the reciprocal aggression call experiments revealed no effect of affect intensity on the response behaviour to the test stimuli (Binomial test, n = 6, x = 2, p = 0.34).

### Discrimination of affect intensity in response call stimuli?

The number of response call stimuli needed for habituation did not differ between experiments (“weak to strong” 4.5 (3 – 7) stimuli, “strong to weak” 5 (3.75 – 6) stimuli, and “strong to low frequency” 5 (4 – 6.25) stimuli; Friedman Anova, n = 12, df = 2, chi^2^ = 1.409, p = 0.49), but varied significantly with the number of experiments performed (first experiment 6.5 (5 – 10.5) stimuli, second experiment 4 (3 – 5.25) stimuli, third experiment 4.5 (4 – 5) stimuli; Friedman Anova, n = 12, df = 2, chi^2^ = 7.955, p = 0.019), indicating a long-term memory effect to consecutive response call experiments. Figure 2 shows the number of bats reacting in the reciprocal experiments (C, experiment “weak to strong”; D, experiment “strong to weak”) and the control experiment (E, experiment “strong to low frequency”). The number of bats reacting to the second to last habituation stimulus, the last habituation stimulus, the test stimulus and the re-habituation stimulus differed significantly in experiment “weak to strong” (Cochran’s Q test, n = 12, df = 3, Q = 8.81, p = 0.032). After habituation to stimuli of low affect intensity, a significant number of bats responded to the test stimulus of high affect intensity (subset comparison of Cochran’s Q test, n = 12, df = 1, Q_diff-test_ = 5.45, p < 0.02). In contrast, the bats transferred the habituation to the test stimulus in the reciprocal experiment (Cochran’s Q test, n = 12, df = 3, Q = 2.2, p = 0.53) indicating an asymmetry in response behaviour. This suggests that the novelty of the test stimulus is not sufficient to categorise it as a separate class.

The direct comparison of responses for bats reacting differently to the two test stimuli (n = 5) in the reciprocal response call experiments confirmed the asymmetry in response behaviour: these bats reacted exclusively, and significantly, to the test stimulus of high affect intensity (Binomial test, n = 5, x = 0, p = 0.031).

This asymmetry (see Figure 2C, D) cannot be attributed to an over-habituation by response call stimuli of high affect intensity in general, since the bats discriminated the control stimulus in experiment “strong to low frequency” (Figure 2E; Cochran’s Q test, n = 12, df = 3, Q = 9.0, p = 0.029; subset comparison, n = 12, df = 1, Q_diff-test_ = 6.0, p < 0.02).

Looking times decreased significantly from the first habituation stimulus to the second to last, and last, habituation stimulus (Fisher permutation tests, n = 12, p < 0.01 for all response call experiments) for all bats including those individuals, for which the video analysis had revealed a body turn to the second to last, or last, habituation stimulus.

**Figure 3:**
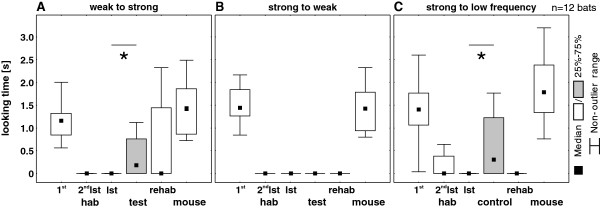
**Duration of body turn in response call experiments.** Duration of body turn (looking time [s]) to response call stimuli of twelve bats (medians, inter-quartiles and non-outlier range) to the first habituation stimulus (1^st^ hab), the second to last habituation stimulus (2^nd^ lst hab), the last habituation stimulus (lst hab), the test stimulus (test/ control, highlighted in grey), the re-habituation stimulus (rehab), and the mouse distress call (mouse) is given for experiments “weak to strong” **(A)**, “strong to weak” **(B)** and “strong to low frequency” **(C)**. An asterisk indicates a significant difference in looking time between the last habituation stimulus and the test stimulus.

In experiment “weak to strong”, the looking time to the test stimulus was significantly longer than that to the last habituation stimulus (Fisher permutation test, n = 12, p = 0.031; Figure 3A). For the six individuals that responded to the test stimulus, the average looking time was 1.3 s to the first habituation stimulus, 0.0 s to the last habituation stimulus and 0.8 s to the test stimulus, indicating discrimination, although the bats showed a shorter looking time than to the first habituation stimulus. In the reciprocal experiment, the average looking time to the first habituation stimulus amounted to 1.4 s, the looking times (0.0 s) to the last habituation stimulus and the test stimulus did not differ significantly (Fisher permutation test, n = 12, p = 0.5; Figure 3B), i.e. habituation was transferred to the test stimulus.

In experiment “strong to low frequency”, the bats reacted significantly longer to the control stimulus (Fisher permutation test, n = 12, p = 0.016; Figure 3C) than to the last habituation stimulus. Here, the average looking time for the six individuals reacting to the control stimulus amounted to 1.6 s to the first habituation stimulus, 0.0 s to the last habituation stimulus and 1.5 s to the control stimulus, i.e. looking times of these individuals to the first habituation stimulus and the control stimulus were indiscriminable.

The looking times for the re-habituation stimuli did not differ significantly from those for the last habituation stimuli in experiments “strong to weak” (Fisher permutation test, n = 12, p = 0.125, Figure 3B) and “strong to low frequency” (Fisher permutation test, n = 12, p = 0.25; Figure 3C), and revealed a release from habituation in experiment “weak to strong” (Fisher permutation test, n = 12, p = 0.031; Figure 3A).

## Discussion

The present playback experiments exploring the effects of conspecific vocalisations differing in affect intensity on auditory classification in a bat model provided different results for the two call types tested, aggression, and response, calls. In the pre-test, stimuli evoked statistically indiscriminable distraction effects, irrespective of call type, and affect intensity. This similar, initial interest in the playback stimuli renders it unlikely that the methodical differences in creating the playback stimuli account for the different behavioural responses in the habituation-dishabituation experiments. Rather, habituation to a given call type and affect intensity created a specific emotional pre-experience, which modulated response behaviour markedly. For aggression calls, the bats needed more stimuli to habituate, and the order of the respective experiments had no effect on the number of stimuli needed for habituation. Thus, the bats remained highly susceptible to aggression call stimuli despite repeated pre-experience. In the reciprocal experiments, we found no evidence for a discrimination of aggression call stimuli according to affect intensity. For response calls, the bats needed fewer stimuli for habituation, yet the number of stimuli needed for habituation decreased with the number of experiments performed, suggesting a long-term memory effect. In the reciprocal experiments, test stimuli elicited asymmetric response behaviour, providing evidence for stimulus classification according to affect intensity, as well as for stimulus evaluation according to pre-experience. In view of the different effects of aggression and response calls, we shall relate the above results to the hypotheses proposed in the introduction, separately for each call type. Then, we shall discuss how the perception of affect intensity can be accounted for by a common neural mechanism across call types.

### Perception of aggression calls

In contrast to our initial hypothesis (I), bats did not classify aggression calls according to affect intensity, i.e. the test stimulus from the opposite affect intensity was not reliably detected in the reciprocal experiments.

Yet, the aggression call experiments are revealing with respect to the perception of this physically salient call type. A common functional hypothesis ascribes an adaptive value to vocalisations containing clicks, noise, or non-linearities, in contexts of imminent threat: as noisy stimuli are less predictable than tonal ones, and are assumed to act by directly accessing low-level brain stem mechanisms [11], it has been postulated that they are harder to habituate to (e.g. [11,12,46,47]). Indeed, single presentations of stimuli with noisy segments elicited increased responsiveness compared to stimuli without noisy segments and this was interpreted as an effect of higher unpredictability (e.g. [48,49]). However, effects of stimulus noisiness on habituation itself have barely been addressed; an exception are experiments on tree shrews, for which only 45% of the subjects have been reported to habituate to noisy chatter calls, in contrast to 85% habituating to tonal squeaks [35].

The present results confirm the above functional hypothesis, as the bats needed significantly more stimuli to habituate to the aggression calls containing click trains than to the response calls consisting of tonal syllables (for detailed call descriptions see [40]). In addition, although the bats habituated significantly to repeatedly presented aggression call stimuli of a given affect intensity, it is questionable whether they habituated to the affect intensity of the stimuli. Some bats responded to the novel test stimulus and the following re-habituation stimulus, irrespective of affect intensity, suggesting that they had rather habituated to the limited set of previously heard stimulus exemplars. Thus, in contrast to the study with tree shrews, in which the subjects that had habituated to noisy chatter calls transferred this habituation to a novel stimulus of the same affect intensity [35], any novel aggression call stimulus was able to grab the bats’ attention again. In sum, the perception of novel aggression calls proved to be robust with respect to pre-experience in our experiments emphasising the high ecological relevance of this call type.

### Perception of response calls

The behaviour of the bats in the response call experiments corresponded to our hypotheses as follows. A test stimulus of high affect intensity presented after habituation to low affect intensity stimuli was discriminated as belonging to a novel class. In the reciprocal experiment, the pre-experience with stimuli of high affect intensity masked novelty detection for the test stimulus of low affect intensity. This response asymmetry showed that test stimuli were not classified according to “novelty”, but implied a classification of stimuli according to affect intensity in experiment “weak to strong”, confirming hypothesis (I). Moreover, the effect of emotional pre-experience on stimulus classification by the bats contradicts hypothesis (II), and confirms hypothesis (III). A control stimulus with a mere frequency shift relative to the habituation stimuli was classified as novel in contrast to the prediction of hypothesis (IV). Thus stimulus salience, reflecting both the affect intensity of the stimulus and acoustic pre-experience, rather than the acoustic structure of the stimulus alone, determined the response behaviour in the reciprocal experiments.

To sum up, pre-experience was an essential factor determining the perception of response calls. According to Owren and Rendall [11], tonal stimuli shaped by vocal tract properties, such as the response calls, may modulate the behaviour of the listener via a learned affect. This implies that the perception of tonal stimuli is more susceptible to pre-experience than that of noisy stimuli which is in agreement with the present findings. In contrast to aggression call perception, pre-experience was not only reflected in the habituation to series of repeatedly presented stimulus exemplars, but also in long-term memory effects across successive experiments and, crucially, in the asymmetric response behaviour to test stimuli in the reciprocal experiments.

These pronounced effects of pre-experience shaping the auditory perception of affect intensity corresponded to those reported for humans. In humans, perceived affect intensity for affective, non-linguistic vocalisations was lower after adaptation to stimuli of the same affect [50]. Likewise, humans showed a neural response asymmetry depending on the affect intensity of the standard stimuli in an oddball paradigm experiment [31]. The present results in bats provide evidence that this evaluation of emotional pre-experience is not specifically human, but may in fact constitute a universal mammalian trait.

### Mechanisms underlying perception of affect intensity in aggression and response calls

It is a central finding of the present study that bats perceive affect intensity in aggression and response calls differently. To account for this difference, indicated by the distraction of the experimental subject from the on-going task, the following section focuses on neural processing mechanisms controlling the re-direction of attention and their interaction with emotional acoustic stimuli of distinct structure.

The current multi-stage models (e.g. [51,52]) of emotional prosody perception based on neuro-imaging studies in humans (for review, see [53]) propose that the first stage of processing provides a parallel extraction of basic acoustic features. In the second stage, these acoustic properties are integrated into an emotional percept. Finally, in the third stage, this emotional percept is subject to evaluation processes. Pre-experience creating a specific contextual and individual significance may affect all three stages via a central feedback. The study of event-related potentials (for review, see e.g. [54]) disclosed that both, the neural representations of acoustic features (corresponding to the first stage) and the percept present in the sensory memory (belonging to the second stage) created by repeated stimulus presentations are able to evoke the involuntary re-direction of attention via a bottom-up “attention call” (sensu Näätänen et al. [54]). The sensory-memory percept represents derived qualities, such as affect intensity, which are the result of a pre-attentive binding of sufficiently homogeneous stimulus features [55]. This bottom-up formation of the sensory memory cannot be replaced by a top-down controlled focus on certain features [56]. In contrast to sensory memory formation, the “attention calls” are subject to top-down central feedback via the modulation of excitability for basic feature extraction and via the evaluation of the output of the sensory memory, respectively. As a consequence of this central feedback, the “attention call” reflecting auditory change detection in sensory memory need not necessarily re-direct attention to a deviant acoustic stimulus. This may constitute the functional basis of experience-dependent response asymmetries [57].

Comparable mechanisms may govern the re-direction of attention in response to acoustic stimuli throughout mammals (e.g. [58-61]). Thus, the above model integrating the effects of vocalisation structure and auditory pre-experience may explain the response behaviour of the bats to stimuli differing in affect intensity.

First, the Näätänen et al. [54] model predicts that a sensory-memory percept of affect intensity is only formed if a pre-attentive binding of features characteristic for affect intensity takes place. Without a sensory-memory percept of affect intensity, the acoustic features of the stimulus are the only relevant factor acting as “attention call” for the behavioural response. The present results suggest that habituation with aggression calls did not result in the formation of a sensory-memory percept of affect intensity. Instead the acoustic features of novel aggression calls were sufficient to elicit a response in the present study, corresponding to the prediction of the model.

Second, the model accounts for the observed, experience-dependent response asymmetries to response calls: the discrimination of stimuli according to affect intensity suggests that the bats integrated stimulus features into a sensory-memory percept of affect intensity during habituation, while the fact that the bats responded to an increase, but not a decrease, of affect intensity may reflect the evaluation of emotional salience.

Third, the model predicts that the sensory-memory percept of affect intensity is only modified by a novel stimulus, if its acoustic features are in the range characteristic for the sensory memory of affect intensity. Deviant features outside this range, however, may directly elicit the “attention call”, without involvement of the sensory memory of affect intensity. This accounts for the discrimination of the control stimulus in the response call experiments. The fundamental frequencies of the control stimulus were about 6 kHz lower than those of the habituation stimuli, a frequency shift in the inter-individual range of response calls, however outside the range of affect-induced frequency shifts which differ by only 0.8 (0.6 - 1.1) kHz (according to a re-analysis of original data of Bastian and Schmidt [40]) within the individual.

Finally, the Näätänen et al. [54] model explains how the modification of the sensory-memory percept affects the processing of a subsequent stimulus. For the expected response to the re-habituation stimulus in our paradigm, the model predicts a release from habituation only if the sensory memory of affect intensity is involved in the “attention call”, and if, in addition, the evaluation of the test stimulus has changed the sensory-memory percept. Indeed, the bats responded to the re-habituation stimulus only in experiment “weak to strong”, but not in experiment “strong to low frequency”, in which the response to the control stimulus could not be based on its affect intensity. The evaluation of the test stimulus in experiment “weak to strong” may have modified the sensory-memory percept established during habituation with stimuli of low affect intensity, resulting in the release from habituation to the re-habituation stimulus. This explanation challenges the use of re-habituation stimuli to control for habituation level (see [62]) in mammals. If the increased emotional salience of the test stimulus modifies the sensory-memory percept established by pre-experience, this change will create the observed release from habituation, and the re-habituation stimulus is unsuitable to control for habituation level. Indeed, animals responded to the re-habituation stimulus also in other playback experiments with test stimuli from situations of increased emotional salience, namely the gathering of the own social group [42], or the herding of females [34].

## Conclusions

In sum, the present study revealed that call structure and auditory pre-experience determine the response behaviour to acoustic stimuli of different affect intensity, and provided evidence for the evaluation of affect intensity in a non-human mammal. This evaluation indicates that the perception of emotions in the voice of conspecifics may be a shared mammalian trait, as it is present in a taxon evolutionarily remote from humans.

## Methods

### Animals and keeping conditions

Twelve adult bats (7 females and 5 males) originating from the Pannian Hill complex (N 9.97898 E 77.96205) near Madurai, South India, were kept in a flight room (3.1 m × 2.4 m × 2.2 m) at the campus of the Madurai Kamaraj University, Madurai, between the end of November 2009 and the beginning of April 2010. Bats were labelled using collars that carried capital letters for identification. The ceiling of the flight room was created by mosquito netting suitable to perch and move around; an open cage (0.8 m × 0.8 m × 0.8 m) positioned on a table gave the bats an opportunity to withdraw. We maintained a natural light–dark cycle. The bats were fed daily after sunset during the experiments with dead frogs of different common species of the genus *Rana*. Additionally, frogs and insects, mostly dragonflies, were provided in the flight room after the experiments. The diet was enriched with vitamins (Multibionta, Merck GmbH, Darmstadt, Germany) injected subcutaneously into dead frogs. Water was provided ad libitum in the flight room. Each bat was released at the capture site as soon as data acquisition was completed.

The present research was cleared for implementation by the Internal Research and Review Board (IRB), Ethical Clearance (EC), Biosafety and Animal Welfare Committee of Madurai Kamaraj University, Madurai (approval letter dated October 05 2009), and carried out in strict accordance with the current laws of India.

### Playback stimuli

The playback stimuli used in the present experiments were based on sound recordings of Bastian and Schmidt [40] made during approach situations resulting in agonistic interactions of bat dyads. Below, we use the term weak, or strong, for calls recorded in situations validated by Bastian and Schmidt [40] as low, or high, intensity level interactions, respectively. Assuming that the median call parameters determined by Bastian and Schmidt [40] for the respective affect intensity represent prototypical calls, we created the stimuli for the present experiments comprising weak aggression calls, strong aggression calls, weak response calls, strong response calls and a control stimulus differing in syllable peak frequencies from strong response calls, as follows.

Weak, and strong, aggression calls from six bats were cut from the validated call recordings and high-pass filtered at 10 kHz (2^nd^ or 4^th^ order Butterworth filter; recording artefacts occasionally occurring at about 90 kHz were removed with an additional bandstop filter) using BatSound Pro (Version 3.31, Pettersson Elektronik AB). The set of weak aggression calls comprised 16 exemplars, the set of strong aggression calls comprised ten exemplars (see examples in Figure 1A, B). The selected calls had durations within the typical inter-quartile range for the respective affect intensity. Each call was embedded in its natural noise floor; signal-to-noise ratios ranged between 30 dB and 55 dB. Stimuli were set to equal maximum amplitudes. Median sound pressure levels determined with a measuring amplifier (Brüel & Kjaer type 2209) amounted to 60.4 ± 1.5 dB SPL for weak aggression calls and 63.7 ± 1.7 dB SPL for strong aggression calls at the bat’s position in the playback experiments.

As response calls are typically overlapping with multiple echoes from aggression calls, and recordings frequently contain echolocation calls of other bats, it is problematic to directly use response calls from original recordings for playback. Moreover, in contrast to aggression calls, the response calls carried distinct individual signatures [40] which may interfere with affect discrimination behaviour. Therefore we created synthetic replicas based on the two validated response calls in the sample of Bastian and Schmidt [40] best reflecting the median call parameters for low, and high, affect intensity, respectively. Call syllables were created with the graphic synthesizer of AviSoft-SASLab Pro (Avisoft Bioacoustics, Berlin) and were combined to calls using BatSound Pro. The two synthetic calls (Figure 1C, D) corresponded to the respective natural exemplars (see for example Figure 1E) in frequency-time contours of syllables as well as in the typical frequency-time course and amplitude-time course across syllables. In addition, these synthetic calls reflected the median parameter values for the frequency position and harmonic composition of syllables, as well as for syllable numbers given in Bastian and Schmidt [40]. Based on this median weak, and strong, response call, we synthesised a set of eight additional response call stimuli, which represented the inter-quartile variability of the validated response call sample of Bastian and Schmidt [40] in syllable duration, inter-syllable interval and fundamental frequency (for a detailed stimulus description see electronic supplement Additional file 1). In addition, we created a control stimulus, identical to the median strong response call except for syllable peak frequencies which were shifted downwards by about 6 kHz, a difference within the range of inter-individual variability (Figure 1F). Stimuli had equal maximum amplitudes resulting in median sound pressure levels of 58.6 ± 0.2 dB SPL for weak response calls and 61.4 ± 0.2 dB SPL for strong response calls.

The duration of all stimuli amounted to 270 ms; call onsets occurred 10 ms after stimulus onset. The stimuli corresponding best to the median calls, and the control stimulus, served as test stimuli. All other stimuli were used for habituation.

### Experimental setup

In the experimental room (3.1 m × 2.4 m × 2.2 m), a V-shaped perch (0.15 m × 0.1 m) was positioned at a height of 1.9 m in the middle of the short side of the room at a distance of 0.2 m from the back wall. A loudspeaker (quadral ribbon tweeter 923108, frequency response linearly (± 3 dB) decreasing by 12 dB between 9 and 90 kHz) was positioned at a distance of 1 m to the left, or right, side of the perch at the height of the bat’s ears. A video camera (Sony DCR-HC90E) facing the perch positioned at a distance of 2.0 m from, and at the same height as, the perch, recorded the bat and an LED, which was mounted behind the perch to indicate the presentation of a stimulus. We used the night shot function during recording. Below the video camera, a feeder, which could be opened and closed with a thread, was installed at a height of 0.60 m. An infrared illuminator (Abus Ecoline TV 6700) below the feeder pointed towards the perch and provided extra light. The experimenter sitting on the floor behind the feeder operated the video camera and the control laptop (Toshiba Satellite A60), which was weakly illuminated by a red light bulb. Custom made software (M. Großbach) controlled stimulus output which was fed via a soundcard (NI DAQCard-6062E, output rate 200 kHz, DAC resolution 12 bit), and a custom made amplifier to the loudspeaker.

### Training of bats and experimental procedure

Before playback experiments started, each bat was individually trained to hang at the tip of the V-shaped perch, wait for the opening of the feeder, catch a frog and then return to the perch for eating. Typically, a playback experiment started after the first food uptake when the bat was looking directly towards the feeder and was thus optimally positioned to the loudspeaker. Daily feeding sessions without playbacks were occasionally interspersed with the playback experiments to avoid over-habituation to the experimental procedure; experiments were separated by at least one night and conducted in random order. Before performing any habituation-dishabituation experiments, we ran a pre-test, in which the four test stimuli used in the reciprocal experiments and the response call control stimulus were played back once in random order to compare their spontaneous distraction effects on the bats. The interval between two stimulus presentations amounted to at least five minutes.

The habituation-dishabituation experiments were performed using an adapted version of the paradigm originally developed by Eimas et al. [32], in which subjects indicated a stimulus categorisation by reacting to a stimulus of a given class after habituating to stimuli of another class. During habituation, different exemplars of a given call type and affect intensity (hab) were presented in random order until the experimenter observed no reaction to two consecutive stimuli. Then, we presented the respective test stimulus of other affect intensity (test), or the frequency shifted control stimulus (control). The test/control stimulus was followed by the re-habituation stimulus (rehab) randomly picked from the set of habituation stimuli, a procedure proposed as a control for habituation level (see [62]).

Stimuli were played back every 20 s. At the end of the experiment, we controlled for overall motivation by playing back one of 14 mouse distress calls (mouse; for definition see [63]) from a sound library (Institute of Zoology, University of Veterinary Medicine Hannover Foundation). Since mice are part of the natural prey spectrum of *M. lyra*, these calls are highly attractive acoustic stimuli. If a bat did not react to the first or second habituation stimulus, or to the mouse call, the experiment was repeated on another day.

### Video analysis and statistical analysis

All experiments were videotaped and videos were analysed while muted in order to keep the rating person blind to the stimuli the bat heard. Periods of stimulus presentation were indicated via the synchronised LED signal. The response behaviour of the bat consisted of a turning of the body away from the feeder. We defined each rotation of the bat’s elbows around the apical-caudal body axis as reaction.

To analyse distraction effects to the five test/control stimuli in the pre-test, we performed a frame-by-frame analysis (Interact 8.0, Noldus, 25 frames/s). In analogy to studies in primates (e.g. [33,37]), we measured the “looking time” which we defined as the duration from the onset of the turning reaction to the end of the turning movement towards the feeder. We compared the looking times for the different stimuli and for the order of their presentation using a multiple comparison test (Friedman Anova).

To analyse the habituation-dishabituation experiments, two persons first conducted a blind rating with Windows Media Player (Windows XP Professional). The rating, using the categories “reaction” and “no reaction”, resulted in a 90% agreement between observers, corresponding to a substantial inter-observer reliability (κ = 0.8, [45], p. 450 ff]).

The number of stimuli needed for habituation was compared by a pair-wise comparison (aggression call stimuli, Fisher permutation test, see ([64], p. 85), implemented in GNU Octave Version 3.2.4, [65]), or by a multiple comparison (response call stimuli, Friedman Anova). To assess global effects of a repeated presentation of aggression call, and response call, stimuli, we compared the number of stimuli needed for habituation as a function of experimental order (first and second experiment with aggression call stimuli, Fisher permutation test; first, second and third experiment with response call stimuli, Friedman Anova), respectively.

To test for habituation, we compared the number of bats reacting to the first habituation stimulus, the second to last habituation stimulus, and the last habituation stimulus using Cochran’s Q test. To test whether the test stimulus and the habituation stimuli were perceived as different classes, we performed a Cochran’s Q test comparing the number of animals reacting to the second to last habituation stimulus, the last habituation stimulus, the test stimulus, and the re-habituation stimulus. If this test detected a significant difference, we applied the subset comparison supplement of Cochran’s Q test (test statistic Q_diff_, see ([45], p. 171) to differentiate between the reaction to the test stimulus versus the reactions to the second to last habituation, the last habituation and the re-habituation stimuli (Q_diff-test_), or between the last habituation stimulus versus the second to last habituation stimulus, the test and the re-habituation stimulus (Q_diff-last_).

To test for symmetry of the responses to the test stimuli in the reciprocal experiments, we directly compared the number of bats changing their behaviour between the experiments by using one-sided Binomial tests (see [64], p. 64).

If a significant number of bats reacted to the test stimulus in at least one habituation-dishabituation experiment with a given call type, we conducted a frame-by-frame analysis for all experiments with the respective call type and measured the looking time to obtain a quantitative measure of the bats’ behaviour. We tested whether the bats were habituated at the end of the habituation process (first versus second to last, and last, habituation stimulus), whether they showed a dishabituation response to the test stimulus (last habituation versus test stimulus), and whether they were still habituated after test stimulus presentation (last habituation versus re-habituation stimulus) using pair-wise comparisons (Fisher permutation tests; see ([64], p. 85), implemented in GNU Octave Version 3.2.4, [65]). Global significance level was 5% for all tests. Unless otherwise stated, statistical analyses and figures were made in Statistica (Version 6, StatSoft Inc.). Medians and inter-quartiles are given for descriptive statistics. The non-outlier range given in the box-plots comprises data points if their value was smaller than the 75% quartile + 1.5 × inter-quartile range, or larger than the 25% quartile - 1.5 × inter-quartile range, using a standard measure for non-normally distributed data sets.

## Competing interests

The authors declare that they have no competing interest.

## Authors’ contributions

HBK participated in the design of the study and data acquisition, analysed the data and participated in the interpretation of data and the drafting of the manuscript. AKVK and SK participated in study design, data acquisition and revised the manuscript. SK and SS initiated the study. SS financed and mentored the study and participated in its design, the interpretation of the data and the drafting of the manuscript. All authors have read and approved of the final manuscript.

## Supplementary Material

Additional file 1 **Spectral and temporal parameter values of synthesised response call syllables.** In total, we synthesised nine weak response call stimuli comprising nine u-shaped syllables (usy) and ten strong response call stimuli comprising 17 u-shaped syllables differing in peak frequency (peak freq [kHz]) of, duration (dur [ms]) of, and inter-pulse intervals (IPI [ms]) among, u-shaped syllables. Stimulus composition was based on the medians, and the lower and upper quartiles, of the parameter values given in Bastian and Schmidt [40]. Each stimulus (no. 1, 2, 3…) was composed by combining the parameter values of peak frequency (no. 1, 2, 3…), duration (no. 1, 2, 3…) and inter-pulse interval (no. 1, 2, 3…) of the respective column. Click here for file
