# Supplementary material for: Auditory pre-experience modulates classification of affect intensity: evidence for the evaluation of call salience by a non-human mammal, the bat Megaderma lyra
Source: Front Zool. 2013 Dec 16;10:75. doi: 10.1186/1742-9994-10-75 (PMC3866277; doi:10.1186/1742-9994-10-75)
Supplement: Additional file 1 — Spectral and temporal parameter values of synthesised response call syllables. In total, we synthesised nine weak response call stimuli comprising nine u-shaped syllables (usy) and ten strong response call stimuli comprising 17 u-shaped syllables differing in peak frequency (peak freq [kHz]) of, duration (dur [ms]) of, and inter-pulse intervals (IPI [ms]) among, u-shaped syllables. Stimulus composition was based on the medians, and the lower and upper quartiles, of the parameter values given in Bastian and Schmidt [40]. Each stimulus (no. 1, 2, 3…) was composed by combining the parameter values of peak frequency (no. 1, 2, 3…), duration (no. 1, 2, 3…) and inter-pulse interval (no. 1, 2, 3…) of the respective column. [file 1742-9994-10-75-S1.pdf]

ESM 1

Spectral and temporal parameter values of synthesised response call syllables.

In total, we synthesised nine weak response call stimuli comprising nine u-shaped syllables (usy) and ten strong response call stimuli comprising 17 u-shaped syllables differing in peak frequency (peak freq [kHz]) of, duration (dur [ms]) of, and inter-pulse intervals (IPI [ms]) among, u-shaped syllables. Stimulus composition was based on the medians, and the lower and upper quartiles, of the parameter values given in Bastian and Schmidt [40].

Each stimulus (no. 1, 2, 3…) was composed by combining the parameter values of peak frequency (no. 1, 2, 3…), duration (no. 1, 2, 3…) and inter-pulse interval (no. 1, 2, 3…) of the respective column.

weak response call stimuli															
**stimulus no.**	**1, 2, 3**	**1, 2, 3**	**1**	**2**	**3**	**4, 5, 6**	**4, 5, 6**	**4**	**5**	**6**	**7, 8, 9**	**7, 8, 9**	**7**	**8**	**9**
**usy no.**	**peak freq [kHz]**	**dur [ms]**	**IPI [ms]**	**IPI [ms]**	**IPI [ms]**	**peak freq [kHz]**	**dur [ms]**	**IPI [ms]**	**IPI [ms]**	**IPI [ms]**	**peak freq [kHz]**	**dur [ms]**	**IPI [ms]**	**IPI [ms]**	**IPI [ms]**
**1**	17.7	4.05	7.71	6.46	4.98	17.7	4.03	7.75	6.55	5	18.2	4	7.72	6.57	5.04
**2**	18.2	3.74	7.71	6.47	5	18.4	3.73	7.86	6.64	4.96	19.1	3.73	7.77	6.55	5.03
**3**	19.1	2.57	7.71	6.5	5.01	20.2	2.58	7.75	6.53	5.03	20.6	2.56	7.71	6.5	5
**4**	19.3	2.71	7.6	6.4	4.89	19.7	2.71	7.68	6.47	4.99	20	2.69	7.75	6.57	5.04
**5**	18.9	2.99	7.68	6.44	4.96	18.9	3.02	7.65	6.42	4.93	19.3	2.99	7.7	6.51	4.97
**6**	18	3.4	7.67	6.45	4.84	18.4	3.44	7.63	6.41	4.96	18.6	3.42	7.76	6.55	4.99
**7**	17.5	2.99	7.71	6.47	4.89	18	3	7.67	6.45	4.96	17.7	3.02	7.72	6.53	4.98
**8**	17.5	2.96	7.68	6.43	4.84	17.1	2.98	7.74	6.52	5.02	17.5	2.96	7.64	6.43	9.96
**9**	17.2	3.07	-	-	-	17.2	3.1	-	-	-	17.3	3.1	-	-	-

strong response call stimuli																
**stimulus no.**	**1, 2, 3**	**1, 2, 3**	**1**	**2**	**3**	**4, 5, 6**	**10**	**4, 5, 6, 10**	**4**	**5, 10**	**6**	**7, 8, 9**	**7, 8, 9**	**7**	**8**	**9**
**usy no.**	**peak freq [kHz]**	**dur [ms]**	**IPI [ms]**	**IPI [ms]**	**IPI [ms]**	**peak freq [kHz]**	**peak freq [kHz]**	**dur [ms]**	**IPI [ms]**	**IPI [ms]**	**IPI [ms]**	**peak freq [kHz]**	**dur [ms]**	**IPI [ms]**	**IPI [ms]**	**IPI [ms]**
**1**	18	2.67	6.23	4.88	3.66	18.5	12	2.66	6.12	4.88	3.64	20.1	2.69	6.15	4.89	3.65
**2**	19.2	3.4	6.23	4.83	3.66	19.8	13.1	3.4	6.11	4.91	3.64	20.8	3.36	6.16	4.89	3.67
**3**	20	3.73	6.29	4.94	3.73	21	15	3.73	6.19	4.91	3.71	22	3.71	6.08	4.8	3.6
**4**	19.5	3.75	6.25	4.92	3.71	20.2	14.7	3.77	6.09	4.89	3.59	21.6	3.77	6.18	4.88	3.62
**5**	19.9	3.25	6.23	4.9	3.67	20.4	14.7	3.28	6.08	4.88	3.63	21.7	3.25	6.2	4.93	3.71
**6**	19.9	3.48	6.29	4.94	3.74	20.8	14.8	3.47	6.3	4.79	3.81	21.9	3.46	6.13	4.87	3.64
**7**	19.7	3.26	6.25	4.92	3.7	20.6	14	3.25	6.16	4.83	3.67	21.6	3.24	6.13	4.87	3.62
**8**	19.3	3.9	6.27	4.92	3.71	19.9	13.9	3.92	6.17	4.88	3.69	21	3.88	6.2	4.94	3.71
**9**	18.9	3.01	6.25	4.91	3.69	19.7	13.7	3.01	6.14	4.88	3.65	20.8	3.02	6.14	4.89	3.64
**10**	18.2	3.75	6.21	4.83	3.62	19.1	13	3.73	6.26	4.83	3.77	20.2	3.73	6.21	4.95	3.71
**11**	18.1	3.81	6.25	4.88	3.69	18.7	13.1	3.83	6.18	4.84	3.71	20.2	3.81	6.18	4.92	3.67
**12**	17.7	4.29	6.24	4.88	3.67	18.2	12.4	4.29	6.17	4.94	3.65	19.5	4.26	6.13	4.86	3.6
**13**	17.7	4.01	6.3	4.96	3.74	18	12	4.04	6.09	4.89	3.61	19.5	4.01	6.12	4.84	3.62
**14**	17.3	4.05	6.25	4.88	3.67	17.6	11.9	4.05	6.17	4.87	3.69	18.7	4.06	6.22	4.92	3.73
**15**	17.1	3.75	6.27	4.92	3.71	17.7	11.3	3.76	6.19	4.88	3.69	18.9	3.76	6.17	4.88	3.69
**16**	16.6	3.06	6.2	4.86	3.67	17.2	10.7	3.06	6.17	4.92	3.67	17.8	3.09	6.16	4.86	3.65
**17**	16.1	3.45	-	-	-	16.9	10.4	3.48	-	-	-	17.7	3.46	-	-	-

Title: Auditory pre-experience modulates classification of affect intensity: evidence for the evaluation of call salience by a non-human mammal, the bat *Megaderma lyra*

Journal name: BMC Biology

Authors: Hanna B Kastein[1], AK Vinoth Kumar[2], Sripathi Kandula[2] and Sabine Schmidt[1]

[1]Institute of Zoology, University of Veterinary Medicine Hannover Foundation

Bünteweg 17, 30559 Hannover, Germany

[2]School of Biological Sciences, Madurai Kamaraj University

625021 Madurai, India

e-mail: sabine.schmidt@tiho-hannover.de
